# Diagnostic and interventional circulating biomarkers in nonalcoholic steatohepatitis

**DOI:** 10.1002/edm2.177

**Published:** 2020-08-28

**Authors:** Monica A. Tincopa

**Affiliations:** ^1^ Division of Gastroenterology and Hepatology Department of Internal Medicine University of Michigan Ann Arbor Michigan USA

**Keywords:** biomarkers, disease staging, hepatic fibrosis, NAFLD, NASH

## Abstract

**Introduction:**

In the setting of the obesity epidemic, nonalcoholic fatty liver disease (NAFLD) has become one of the most prevalent forms of chronic liver disease worldwide. Approximately 25% of adults globally have NAFLD which includes those with NAFL, or simple steatosis, and individuals with nonalcoholic steatohepatitis (NASH) where inflammation, hepatocyte injury and potentially hepatic fibrosis are found in conjunction with steatosis. Individuals with NASH, particularly those with hepatic fibrosis, have higher rates of liver‐related and overall mortality, making this distinction of significant clinical importance. One of the core challenges in current clinical practice is identifying this subset of individuals with NASH without the use of liver biopsy, the gold standard for both diagnostics and staging disease severity. Identifying noninvasive biomarkers, an accurately measured and reproducible parameter, would aide in identifying patients eligible for NASH pharmacotherapy clinical trials and to help tailor intensity of monitoring required.

**Methods, Results and Conclusions:**

In this review, we highlight both the currently available and novel diagnostic and interventional circulating biomarkers under investigation for NASH, underscoring their accuracy and limitations relevant to our patient population and current clinical practice.

## Graphical abstract

One of the core challenges in NASH is the ability to accurately diagnose and stage individuals using non‐invasive methods. In this review, we highlight both the currently available and novel diagnostic and interventional circulating biomarkers under investigation for NASH, underscoring their accuracy and limitations relevant to our patient population and current clinical practice.

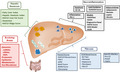

## 1 INTRODUCTION

Nonalcoholic fatty liver disease (NAFLD) has become one of the most prevalent forms of chronic liver disease with a global prevalence of approximately 25% among adults.^1^ NAFLD is the broad umbrella term that encompasses the spectrum of FLD. Histologically, NAFLD is categorized into nonalcoholic fatty liver (NAFL) or nonalcoholic steatohepatitis (NASH).^2^, ^3^, ^4^ To meet diagnostic criteria for NAFL, individuals must have ≥5% hepatic steatosis without evidence of hepatocellular injury. Alternatively, NASH is defined by the presence ≥5% hepatic steatosis with lobular inflammation and hepatocyte injury (ballooning) with or without hepatic fibrosis.^2^ It is estimated that approximately 20% of individuals with NAFLD have NASH.^1^, ^2^, ^5^ Clinical practice guidelines from both the American and European liver societies currently recommend liver biopsy as the gold standard for diagnosing and staging NASH.^2^, ^6^ Enrolment in NASH clinical trials and definition of therapeutic response to novel pharmacologic agents for NASH are also largely defined using histologic criteria.^7^ Inclusion criteria for clinical trials generally include fibrosis stage of ≥F2 on biopsy. Primary outcomes assessing response to novel treatment agents are typically defined using changes in the NAFLD Activity Score (NAS) paired with stability or improvement in fibrosis.^7^, ^8^ There are several notable limitations in liver biopsy including concerns over sampling error and interrater reliability.^9^ In addition, both patients and clinicians are often hesitant to pursue biopsy due to its invasive nature with potential for clinical complications including severe bleeding and rarely death.^10^ As a result, liver biopsy is infrequently obtained in clinical practice for diagnosis and staging of NASH. In real‐world clinical practice, providers often use a combination of noninvasive serum tests, imaging results and endoscopic findings to arrive at a personalized diagnosis and risk stratification for an individual patient.

The clinical differentiation of NAFL vs NASH is important given the distinct natural disease course for these two subsets of NAFLD. Individuals with NASH are at risk for developing advanced fibrosis and cirrhosis and therefore have higher overall and liver‐related mortality.^2^, ^11^, ^12^, ^13^ NASH patients have also been noted to have significantly higher rates of cardiovascular disease and multiple types of cancer in addition to hepatocellular carcinoma (HCC).^13^, ^14^ Recent studies have highlighted the significant clinical implications of fibrosis stage beyond the impact of NASH itself. Individuals noted to have even early stages of fibrosis were found to have significantly increased risk for liver‐related morbidity and mortality.^15^, ^16^, ^17^ Accordingly, a focus on identifying and monitoring fibrosis stage may have more of a clinical impact than differentiating NAFL from NASH.

**FIGURE 1:**
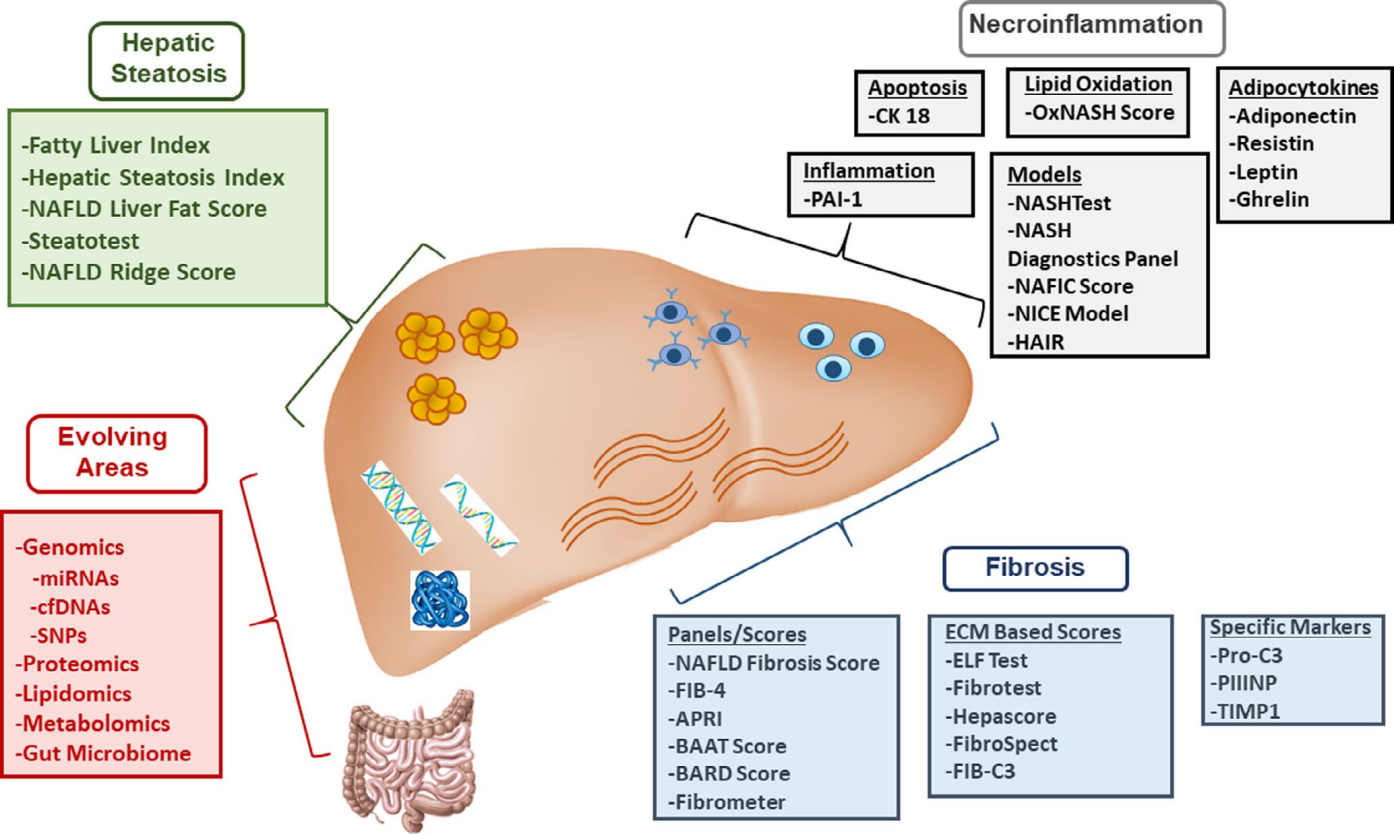
Summary of categories of circulating biomarkers in NASH. Overview of the main categories of circulating biomarkers in NASH with summary of specific biomarkers of interest within each category. APRI, AST to platelet ratio index; cfDNA, cell‐free circulating DNA; ELF, enhanced liver fibrosis; FIB‐4, fibrosis 4; miRNAs, microRNA; NAFLD, nonalcoholic fatty liver disease; PAI, plasminogen activator inhibitor 1; PIIINP, N‐terminal type III collagen propeptide; Pro‐C3, C‐terminal cleavage site of N‐terminal type II collagen propeptide; SNP, single nucleotide polymorphism; TIMP1, tissue inhibitor of metalloproteinases 1

Notably, there are heterogeneous rates of disease progression across individuals, making management of NASH challenging.^18^ Given that a diagnosis of NASH and fibrosis stage has been clearly linked to risk of clinical outcomes and eligibility for and definition of response to emerging pharmacotherapy, there is a significant unmet need to identify noninvasive diagnostic and interventional circulating biomarkers in NASH. By providing accurate, measurable and reproducible markers to diagnose and monitor NASH activity and fibrosis stage, noninvasive biomarkers will enable us to evaluate risk factors for disease progression and identify patients for pharmacotherapy. Interventional biomarkers are of particular interest as these parameters can assist in monitoring response to treatment. There are multiple significant challenges to identifying accurate diagnostic and interventional circulating NASH biomarkers. These challenges emerge due to the heterogeneous and nonlinear rates of disease progression in NASH and uncertainties in the highest yield parameters for monitoring risk of clinical outcomes. In this article, we summarize the currently available and novel investigative diagnostic and interventional circulating biomarkers in NASH to highlight their current potential role in clinical practice and outline possibilities for future care (Figure 1).

## 2 ASSESSMENT OF HEPATIC STEATOSIS

In order to meet diagnostic criteria for NAFLD, an individual must have ≥5% steatosis on histology or ≥5.5% intrahepatic triglyceride content by MRI.^2^ There are several noninvasive circulating biomarkers that have been assessed to evaluate degree of hepatic steatosis and are outlined below.

### 2.1 Clinical decision aides

There are several clinical decision aides to assess for hepatic steatosis that combine laboratory data with clinical features (Table 1). The Fatty Liver Index (FLI) includes triglycerides (TG), gamma‐glutamyltransferase (GGT), body mass index (BMI) and waist circumference (WC) and uses ultrasound (US) as the gold standard reference.^19^ The FLI has moderate performance characteristics with an area under the receiver operating curve (AUROC) of 0.84, sensitivity (Sn) of 84% and specificity (Sp) of 64%. The Hepatic Steatosis Index (HSI) also uses US as the gold standard reference and is comprised of aspartate aminotransferase (AST)/alanine aminotransferase (ALT), sex, BMI and diabetes mellitus (DM).^20^ The HSI has an AUROC 0.81, Sn 93% and Sp 92%. The NAFLD liver fat score uses a more sensitive reference standard, proton magnetic resonance spectroscopy (H‐MRS). It is an algorithm that includes fasting serum insulin, AST, AST/ALT ratios, DM and presence of metabolic syndrome (MetS).^21^ The NAFLD liver fat score had superior accuracy compared to the FLI and HIS with an AUROC of 0.86‐0.87. A decision aide that incorporates more specialized parameters not routinely available in clinical practice is the SteatoTest. This uses the six components of the FibroTest‐ActiTest (total bilirubin, GGT, α‐macroglobulin, haptoglobin, ALT and apolipoprotein AI), total cholesterol, TG, glucose and BMI adjusted for age and sex.^22^ Its diagnostic accuracy is moderate with an AUROC of 0.79‐0.80. Lastly, the NAFLD ridge score applies a machine‐learning algorithm using laboratory results [ALT, high‐density lipoprotein cholesterol (HDL‐C), TG, haemoglobin A1c (HbA1c), and white blood cell count (WBC)] with comorbidity data [hypertension (HTN)].^23^ The NAFLD ridge score also uses H‐MRS as a gold standard and has very good diagnostic accuracy with an AUROC of 0.87 and a negative predictive value (NPV) of 96%.

**TABLE 1:** Noninvasive assessment of hepatic steatosis: Clinical decision aides

Test	Components	Performance characteristics	Reference test	Limitations
Fatty Liver Index^19^	TG, GGT BMI, WC	AUROC: 0.84 Sn 84% Sp 64%	US	Reference test
Hepatic Steatosis Index^20^	AST/ALT, BMI, sex, DM	AUROC: 0.81 Sn 93% Sp 92%	US	Reference test
NAFLD Liver Fat Score^21^	Insulin, AST, AST/ALT DM, MetS	AUROC: 0.86‐0.87 Sn 86% Sp 71%	H‐MRS	Requires fasting
Steatotest^22^	FibroTest‐ActiTest, cholesterol, TG, Glucose BMI, sex, age	AUROC: 0.79‐0.80 Sn 85%‐100% Sp 83%‐100%	Biopsy and original SteatoTest	Cost for proprietary formula
NAFLD ridge score^23^	ALT, HDL‐C, TG, HbA1c, WBC HTN	AUROC: 0.87 Sn 92% Sp 90%	H‐MRS	Research tool

Abbreviations: ALT, alanine aminotransferase; AST, aspartate aminotransferase; AUROC, area under the receiver operating curve; BMI, body mass index; DM, diabetes mellitus; GGT, gamma‐glutamyltransferase; HbA1c, haemoglobin A1c; HDL‐C, high‐density lipoprotein cholesterol; H‐MRS, magnetic resonance spectroscopy; HTN, hypertension; MetS, metabolic syndrome; Sn, sensitivity; Sp, specificity; TG, triglycerides; US, ultrasound; WBC, white blood cell count; WC, waist circumference.

## 3 ASSESSMENT OF NECROINFLAMMATION

The complex underlying pathophysiology of hepatocyte injury involves multiple pathways including but not limited to inflammation, apoptosis, lipid and glucose metabolism and oxidative stress.^24^ Given this, it has been extremely challenging to identify noninvasive biomarkers that accurately capture the degree of necroinflammation in NASH. Table 2 outlines the performance characteristics of the most relevant diagnostic and interventional circulating biomarkers for NASH.

**TABLE 2:** Noninvasive circulating and interventional biomarkers for necroinflammation in NASH

Category	Biomarker	Components	Performance characteristics
Inflammation	PAI‐1^28^, ^29^		NR
Apoptosis	CK 18^32^, ^33^, ^34^, ^35^		AUROC: 0.82‐0.83 Sn: 66%‐78% Sp:82%‐87%
Adipocytokines		Adiponectin, resistin, CK18^43^	AUROC: 0.73‐0.91 Sn: 72% Sp:91%
		Adiponectin, leptin, ghrelin^42^	AUROC: 0.79 Sn: 82% Sp:76%
		FGF21, CK‐18^40^	AUROC: 0.94 Sn: 92% Sp:85%
Lipid Oxidation	oxNASH Score^44^	Linoleic acid:13‐HODE ratio Age, BMI, AST	AUROC: 0.74‐0.83 Sn: 81% Sp:97%
Clinical and Biochemical Models	NASHTest^45^, ^46^	Age, sex, weight, height, TG, cholesterol, a2‐macroglobulin, ApoA1, AST, ALT, haptoglobin, GGT, bilirubin	AUROC: 0.79 Sn: 33% Sp:94%
	NASH Diagnostics Panel^35^, ^48^	CK‐18‐M65, CK18‐M30, resistin, adiponectin	AUROC: 0.91 Sn: 96% Sp:70%
	NAFIC score^49^	ferritin, insulin, type IV collagenS	AUROC: 0.78‐0.85 Sn: NR Sp:NR
	Nice Model^51^	CK‐18‐M30, ALT, MetS	AUROC: 0.83‐0.88 Sn: NR Sp:NR
	HAIR^47^	Insulin resistance, HTN, ALT	AUROC: 0.90 Sn: 80% Sp:89%

Abbreviations: aPAI‐1, activated plasminogen activator inhibitor 1; AUROC, area under the receiver operating curve; FGF21, Fibroblast growth factor 21; GGT, gamma‐glutamyltransferase; HODE, hydroxyoctadecadienoic acid; MetS, metabolic syndrome; Sn, sensitivity; Sp, specificity; TG, triglycerides.

### 3.1 Serum circulating biomarkers of hepatic inflammation

Serum levels of aminotransferases, most commonly ALT, have been frequently applied as routinely available markers of hepatic inflammation in NASH. ALT has consistently been shown to have poor diagnostic accuracy for NASH, with a Sn of 64%, Sp of 75% and an AUROC of approximately 0.60 to detect NASH on liver biopsy in multiple studies.^25^, ^26^, ^27^ Researchers are continuously working to identify serum biomarkers that more accurately capture hepatic inflammation in NASH. Plasminogen activator inhibitor‐1 (PAI‐1) is a serine protease inhibitor that regulates the fibrinolytic system that has been of interest. It has been investigated among patients with biopsy‐proven NAFLD and been shown to be associated with underlying NASH.^28^, ^29^, ^30^, ^31^ Among 273 patients with obesity, PAI‐1 levels were correlated with severity of steatosis, lobular inflammation, hepatocyte ballooning and fibrosis.^28^ Similar findings were noted among patients enrolled in the NASH Clinical Research Network where PAI‐1 was associated with histologic NASH (OR 1.2, 95% CI 1.08‐1.34).^29^

### 3.2 Circulating biomarkers of hepatocyte apoptosis

Cytokeratin 18 (CK‐18) is a major intermediate filament protein in hepatocytes. In the setting of hepatocyte death, CK‐18 has been shown to be released at higher levels in NASH compared to NAFL. CK‐18, including multiple different CK‐18 fragments, has been studied extensively in relationship to NASH.^32^ In meta‐analyses, CK18‐M30 levels had a pooled AUROC of 0.82 (0.76‐0.88) for identifying NASH with a Sn 66%‐78% and Sp of 82%‐87%.^33^, ^34^ Levels of CK18‐M65 had similar accuracy with an AUROC of 0.82.^35^ Interpretation of these studies is complicated by the widely variable optimal cut‐off used to generate the associated Sn and Sp. Numerous models have combined CK18 with other blood‐based parameters and clinical features and demonstrated improved prediction of NASH among individuals with NAFLD.^36^ A model that combines CK18 fragments with C‐terminal cleavage site of procollagen type III N‐terminal peptide (Pro‐C3), acetyl‐high mobility group box 1 and patatin‐like phospholipase domain‐containing protein 3 (PNPLA3) rs738409 had the highest reported accuracy to diagnose NASH with an AUROC of 0.87, Sn 71% and Sp 87%, though these results have not been externally validated.^37^

### 3.3 Adipocytokines

Given that adipocytokines are hypothesized to play a central role in the pathogenesis of NAFL and NASH, these markers have also been the subject of investigation as potential biomarkers for disease severity. Fibroblast growth factor 21 (FGF21) is a hormone‐like growth factor involved in several metabolic processes including lipid metabolism and insulin sensitivity.^38^ FGF21 interacts with other relevant adipocytokines including adiponectin and leptin. Prior studies have shown that chronic exposure to FGF21 leads to increased adiponectin levels, which has prompted investigation of an FGF21 analogue as a potential therapeutic agent for NASH.^39^ A meta‐analysis evaluated the diagnostic value of CK‐18, FGF‐21 or a combination panel to diagnose NASH and noted highest Sn (92%) and Sp (85%) in the combination panel compared to FGF‐21 along (Sn 62% Sp 78%).^40^ The associated AUROC of this combination panel was 0.94 (95% CI 0.92‐0.96) to distinguish NASH from NAFL.^40^ Of note, FGF levels fluctuate throughout the day due to regulation by genes that display circadian rhythm. Its hepatic expression is also highly responsive to food intake.^41^ As a result, this variation in levels throughout the day and FGF‐21 levels as they relate to fasting vs fed state require further investigation. A panel including several adipocytokines (leptin, ghrelin and adiponectin) yielded an AUROC of 0.79 to differentiate patients with NASH from those with NAFL.^42^ Lastly, another panel that included adiponectin, resistin and cleaved CK‐18 had good accuracy in the test group (AUROC 0.91) though this dropped significantly in the validation group (0.73) to assess for NASH.^43^

### 3.4 Circulating biomarkers of oxidative stress

Identifying biomarkers of oxidative stress that correlate with NASH has proven challenging in part due to difficulty in measuring these components in serum and their volatile nature. Plasma levels of 9 and 13‐hydroxyoctadecadienoic acid and 9‐13‐oxo‐octadecadienoic acid, products of free radical‐mediated oxidation of linoleic acid, were shown to be elevated among patients with NASH compared to those with NAFL.^44^ Markers of lipid oxidation are of particular interest given their principal role in pathogenesis of NASH. Lipidomic studies have applied mass spectroscopy to find associations with different biomarkers of lipid oxidation with NASH. The oxNASH score is comprised of linoleic acid:13‐hydroxyoctadecadienoic acid (HODE) ratio with AST, age and BMI.^44^ The oxNASH score provides decent diagnostic accuracy with AUROC ranging from 0.74‐0.83, Sn 81% and Sp 97%.^44^

### 3.5 Clinical and biochemical models

Investigators have aimed to improve predictive accuracy by combining clinical variables with circulating biomarkers to correlate with underlying NASH. In general, this approach has yielded improved performance characteristics with AUROCs ranging from 0.76‐0.80 as outlined in Table 2. The NASHTest combines 13 variables including age, sex, weight, height, TG, cholesterol, total bilirubin, ALT, AST, GGT, fasting glucose, α2‐macroglobulin, haptoglobin and apolipoprotein A.^45^, ^46^ Using this combination of variables, the NASHTest yielded an AUROC of 0.79 to differentiate NAFL from NASH. The HAIR test combines HTN, ALT and insulin resistance to provide a score for risk of NASH.^47^ The reported AUROC for the HAIR test was very good at 0.90. A NASH Diagnostics Panel also has a very good AUROC at 0.91.^35^, ^48^ This panel consists of CK‐18‐M65, CK18‐M30, resistin and adiponectin. Two other models that incorporate clinical and laboratory data to differentiate NAFL from NASH are the NAFIC Score and the Nice Model, both of which have good predictive accuracy as outlined in Table 2.^49^, ^50^, ^51^

## 4 ASSESSMENT OF FIBROSIS

Investigation regarding noninvasive assessment of fibrosis stage in chronic liver disease has been ongoing for many years and initially was focused among individuals with chronic hepatitis C. More recently, these efforts have shifted to focus specifically on individuals with NASH as these tests have varying accuracy across different disease states. There are a broad array of approaches using circulating biomarkers including clinical decision aides that combine clinical data with serum biomarkers as well as individual markers of extracellular matrix (ECM) turnover (Table 3). Given that fibrosis stage has been strongly associated for risk of clinical outcomes and overall mortality in NAFL and NASH, identifying noninvasive methods to accurately stage fibrosis is essential.^52^

**TABLE 3:** Noninvasive circulating and interventional biomarkers for fibrosis in NASH

Biomarker	Components	Diagnostic accuracy
Fibrosis Panels/Scores		
NAFLD Fibrosis Score^34^, ^53^, ^57^	AST/ALT, platelets, albumin Age, BMI, hyperglycaemia	AUROC: 0.77‐0.84 Cut‐off: 0.81; NPV: 78%‐93% Cut‐off: 0.67; PPV: 82%‐90%
FIB‐4 index^54^, ^55^, ^56^, ^57^	AST, ALT, platelets Age	AUROC: 0.80‐0.86 Cut‐off: 1.30; NPV: 90%‐95% Cut‐off: 2.67; PPV: 80%
APRI Score^54^, ^55^, ^56^	AST, platelets	AUROC: 0.73 Cut‐off: 1; NPV: 84% PPV: 37%
BAAT Score^61^	ALT, TG Age, BMI	AUROC: 0.84 Cut‐off: 0; NPV: 100% Cut‐off: 1; PPV: 45%
BARD Score^62^	AST/ALT BMI, DM	AUROC: 0.69‐0.81 Cut‐off: 2; NPV: 95%‐97% PPV: 27%
Fibrometer^63^	AST, ALT, platelets, glucose, ferritin Age, Weight	AUROC: 0.94 Cut‐off: 0.49; NPV: 92% PPV: 88%
ELF test^64^, ^65^	HA, PIINP, TIMP‐1	AUROC: 0.87‐0.90 Cut‐off: −1.45; NPV: 93% Cut‐off: 0.67; PPV: 90%
FibroTest^66^	Bilirubin, GGT, haptoglobin, α2‐macroglobulin, apolipoprotein A	AUROC: 0.85‐0.86 Cut‐off: 0.3; NPV: 98% Cut‐off: 0.7; PPV: 60%
Hepascore^67^	Bilirubin, GGT, α 2‐macroglobulin, HA Age, sex	AUROC: 0.81 Cut‐off: 0.37; NPV: 92% PPV: 57%
FIBROSpect^68^	α 2‐macroglobulin, HA, TIMP‐1	AUROC: 0.85‐0.87 Cut‐off: NPV: 81%‐84% Cut‐off: PPV: 72%‐74%
FIB‐C3^69^	Platelets, Pro‐C3 Age, BMI, DM	AUROC: 0.85‐0.86 Cut‐off: 0.3; NPV: 98% Cut‐off: 0.7; PPV: 60%
Specific fibrosis markers		
Pro‐C3^72^		AUROC: 0.91 Cut‐off: 1.67; NPV: 97% PPV: 56%
PIIINP^71^		AUROC: 0.82‐0.84 Cut‐off: 6.6; NPV: 95% Cut‐off: 11 PPV: 100%
TIMP1^75^		AUROC: 0.74 Cut‐off: NR NPV: NR PPV: NR

Abbreviations: APRI, AST to platelet ratio index; AUROC, area under the receiver operating curve; ELF, enhanced liver fibrosis; NPV, negative predictive value; PIIINP, N‐terminal type III collagen propeptide; PPV, positive predictive value; Pro‐C3, C‐terminal cleavage site of N‐terminal type II collagen propeptide; TIMP1, tissue inhibitor of metalloproteinases 1.

### 4.1 Clinical decision aides

The NAFLD fibrosis score (NFS) is a clinical decision aide computed using platelet count, albumin, AST/ALT and three clinical parameters (age, BMI and glucose intolerance).^53^ The NFS has been demonstrated to have very good performance characteristics for assessing likelihood for advanced fibrosis or cirrhosis (AUROC 0.85, Sn 90%, Sp 60%, NPV 88%, PPV 82%), though it is less helpful in discriminating between lower stages of fibrosis.^34^, ^53^ The Fibrosis‐4 index (FIB‐4) and AST to platelet ratio index (APRI) are two other clinical decision aides to assess for underlying fibrosis that are not specific to NAFLD.^54^, ^55^ FIB‐4 is calculated based on platelet count, AST, ALT and age, whereas APRI requires only platelets and AST. FIB‐4 is thought to have better accuracy for predicting the presence of advanced fibrosis in NAFLD compared to APRI.^56^ Both the NFS and FIB‐4 index are currently recommended by the American Association for the Study of Liver Diseases (AASLD) as useful noninvasive and routinely available clinical decision aides to identify patients who may benefit from subspecialty evaluation given risk of advanced fibrosis.^2^ A meta‐analysis demonstrated that the NFS and FIB‐4 have similar accuracy for detecting advanced fibrosis in NAFLD (Sn 72% vs 32%, Sp 70% vs 96% respectively; AUROC 0.84 for both).^57^ In clinical practice, approximately 30% of patients will have scores that fall in the indeterminate range for these tests, however, which limits their utility in these instances.^58^ There are also limitations in terms of generalizability of the performance characteristics reported in derivation studies to the broader population of patients with NAFLD as these scores were constructed primarily among middle‐aged participants who had undergone liver biopsy.^59^, ^60^

Two additional scores of interest to evaluate degree of fibrosis in NAFLD are the BAAT and BARD scores. The BAAT score is comprised of ALT, TG, BMI and age. For prediction of F0, the BAAT score had an AUROC of 0.86, 0.75 for F2, 0.92 for F3 and 0.81 for F4.^61^ The BARD score includes AST/ALT, BMI and DM and generated an AUROC of 0.81 to differentiate patients with NAFL vs those with more advanced fibrosis.^62^ Lastly, there is Fibrometer which consists of fasting glucose, AST, ALT, ferritin, platelets, age and weight. Fibrometer had one of the highest AUROCs to detect significant fibrosis at 0.94.^63^ Overall, these noninvasive scoring systems to assess degree of fibrosis are most useful for their NPV, but do have notable limitations in terms of their PPV and thus must be applied correctly to patient care in clinical practice.

### 4.2 Serum biomarkers of extracellular matrix turnover

There are several panels that incorporate biomarkers of ECM turnover that have been generated to assess correlation with stage of fibrosis in NAFLD. The Enhanced Liver Fibrosis (ELF) panel contains three matrix turnover proteins [hyaluronic acid (HA), tissue inhibitor of metalloproteinase 1 (TIMP‐1) and N‐terminal procollagen III‐peptide (PIIINP)]. In clinical studies, the ELF panel has been shown to have excellent Sn and Sp (80% and 90%, respectively) with an AUROC of 0.90 when used to predict advanced fibrosis or cirrhosis.^64^, ^65^ The FibroTest incorporates bilirubin, GGT, haptoglobin, α2‐macroglobulin and apolipoprotein A. In clinical studies, FibroTest was also shown to have good performance characteristics to detect advanced fibrosis in NAFLD with an AUROC of 0.88.^66^ The Hepascore incorporates clinical variables in addition to laboratory variables (bilirubin, GGT, HA, a2 macroglobulin, age and sex) to assess for significant fibrosis. Among patients with NAFLD, using a cut‐off of 0.37 yielded an AUROC of 0.81 for the Hepascore to detect advanced fibrosis.^67^ FIBROSpect is another combination panel that is also marketed to assess hepatic fibrosis. FIBROSpect consists of α2‐microglobulin, HA and TIMP‐1. Among a cohort of patients with biopsy‐proven NAFLD, FIBROSpect detected advanced fibrosis with an AUROC of 0.87.^68^ When combined with other routinely available clinical data (platelets, age, BMI, DM), a Pro‐C3 based model was accurate in identifying patients with NAFLD and advanced fibrosis with an AUROC of 0.87, NPV 88% and PPV 84%.^69^ Another model constructed using ECM components of HA, CK18 and TIMP‐1 had excellent performance to predict advanced fibrosis in NAFLD with an AUROC of 0.90, Sn 88% and Sp 84%.^70^

Components of the ECM have also been evaluated in isolation as biomarkers to assess fibrosis stage in NASH. A study evaluating PIIINP using cut‐offs of 6.6 ng/mL and 11 ng/mL yielded a NPV of 95% and PPV of 100% for detecting advanced fibrosis.^71^ Another marker of collagen synthesis, Pro‐C3, has been investigated in isolation among patients with NAFLD to detect advanced fibrosis and demonstrated a high AUROC (0.91) with an NPV of 97% and PPV of 56% .^72^ A study evaluating the predictive capability of TIMP‐1 alone to distinguish individuals with NASH from age‐matched controls yielded an excellent AUROC of 0.97.^73^ TIMP‐1 has had conflicting results for fibrosis staging in NAFLD however.^74^ A recent study noted moderate performance for diagnosing significant fibrosis (AUROC 0.74).^75^

## 5 EVOLVING AREAS OF INTEREST FOR NOVEL BIOMARKERS

### 5.1 Genomics

Accumulating evidence highlights the important interaction between environmental and genetic factors in NAFLD, as reviewed in detail in a recent article by Sookoian et al.^76^ MicroRNAs (miRNAs) are short noncoding RNAs that post‐transcriptionally regulate gene expression. Their role as biomarkers in NASH is evolving, though present data are insufficient to strongly support their use. miR‐122 and miR‐34a have been correlated with disease severity in NASH.^77^, ^78^ Cell‐free DNA (cfDNA) has also been evaluated to assess disease severity in NASH, particularly as it relates to degree of fibrosis.^79^ There have been several studies evaluating the role of single nucleotide polymorphisms (SNPs) to evaluate response to lifestyle or pharmacologic interventions in NAFL and NASH. The SNP rs738409 located on GCKR [patatin‐like phospholipase domain‐containing 3 gene (PNPLA3)] has been identified as a consistent genetic modifier in NAFLD.^80^ PNPLA3 I148M variant has been shown to promote hepatic steatosis and stellate cell activation which in turn leads to inflammation and fibrogenesis.^81^, ^82^It has been investigated as a potentially useful biomarker to identify individuals who are more likely to respond to lifestyle interventions or bariatric surgery.^83^, ^84^ The rs58542926 polymorphism in TM6SF2 has been associated with reduced hepatic capacity to secrete very low‐density lipoprotein and thus has been associated with hepatic steatosis and steatohepatitis. Individuals with the TM6SF2 E167K variant are more susceptible to NASH and appear to have protection against cardiovascular disease.^85^, ^86^ The relationship between TM6SF2 rs58542926 polymorphism and risk of NAFLD‐related fibrosis is unclear, with studies having conflicting results. The rs780094 polymorphism at the glucokinase regulatory gene (GCKR) locus is also associated with an increased risk of NAFL and in one study among a large cohort of Italian patients was also associated with severity of liver fibrosis.^87^, ^88^ A polymorphism in the rs641738 variant of the membrane bound O‐acyltransferase domain‐containing 7 (MBOAT7) gene, which is involved in phosphatidylinositol remodelling, has been associated with increased hepatic fat content, more severe hepatocyte injury, increased risk of fibrosis and HCC.^89^, ^90^ Variation in 17‐beta hydroxysteroid dehydrogenase 13 (HSD17B13) which encodes an enzyme localized in lipid droplets within hepatocytes has been associated with protection against hepatic inflammation and fibrosis in the setting of metabolic dysfunction.^91^, ^92^ Similarly, a gene variation at the protein phosphatase 1 regulatory subunit 3b (PPP1R3B) is thought to potentially protect against hepatic fat accumulation and decreases risk of progressive liver disease in patients at high risk for NASH.^93^, ^94^ Lastly, the rs12979850 polymorphism in the IFNλ3 gene that participates in regulation of innate immunity has been associated with increased hepatic inflammation and fibrosis in patients with NAFLD, particularly in lean NAFLD.^95^, ^96^

Several genetic risk scores have been designed to predict the presence of NASH, NASH with fibrosis and NAFLD‐related HCC. These are reviewed in detail elsewhere by Vespasiani‐Gentilucci et al^97^ A genetic risk score consisting of PNPLA3 rs738409, TMSF2 rs58542926 and Kruppel‐like factor 6 (KLF6_rs3750861) was able to identify individuals at risk for NASH cirrhosis among a larger cohort of patients with NAFLD.^98^ Donati et al reported a significant association between the number of risk alleles (PNPLA3 rs738409, TM6SF2 rs58542926 and MBOAT7 rs641738) and the risk of HCC (OR 1.6 per allele).^89^ Lastly, composite biomarker panel was developed among patients enrolled in the GOLDEN‐505 trial of elafibranor to identify patients at risk of fibrosis progression.^99^ This panel included HgA1c, miR‐34a, YKL40 and a_2_m. The AUROC was 0.82 with Sn 73%, Sp 78%, though cross validation of this model has not been completed as of yet.

### 5.2 Proteomics

Proteomics has been applied to help identify candidate biomarkers in NASH. A group of three priority 1 proteins (complement component C7, insulin‐like growth factor acid‐labile subunit and transgelin 2) were able to correctly categorize NAFLD patients with NASH with F3/F4 with an AUROC of 0.91.^65^

### 5.3 Lipidomics and metabolomics

It is hypothesized that lipotoxicity resulting from hepatic inflammation is a mediator of hepatic fibrosis progression.^100^ Therefore, investigators have applied liquid chromatography and mass spectroscopy to conduct lipidomic profiling to help identify individuals with NASH compared to those with NAFL.^101^, ^102^, ^103^ Evaluation of polyunsaturated fatty acid metabolites, with a specific focus on arachidonic acid (AA)‐derived eicosanoids, in a nested case‐control study (N = 10 NAFL, N = 9 NASH, N = 10 non‐NAFLD) yielded an AUROC of 1.0.^101^ The NASH ClinLipMet score was derived using 318 patients with liver biopsies using a combination of clinical, genetic (PNPLA3 genotype), lipidomic and metabolomics data. This yielded excellent performance with an AUROC of 0.86‐0.88 to identify individuals with NASH.^104^ Further confirmatory studies evaluating lipidomic and metabolomic biomarkers are needed to better establish their role in diagnosis and staging of NASH in order to determine their role in clinical practice.

### 5.4 Gut microbiome

Differences in gut microbiome have been evoked in the pathogenesis and risk of disease progression in NASH. It is hypothesized that intestinal microbiota influence hepatic lipid and bile acid metabolism and also contribute to endogenous alcohol consumption.^105^ A small study of patients with NAFLD characterized microbiota signatures and noted an increase in Bacteroides among patients with NASH and an increase in Ruminococcus among patients with F2‐4 compared to those with no to minimal fibrosis.^106^ Interestingly, this is in contrast to findings of another study where there were lower Ruminococcaceae identified among patients with hepatic fibrosis.^107^ Loomba et al used whole‐genome shotgun sequencing of stool DNA to detect advanced fibrosis among 86 patients with NAFLD. Though not validated as of yet, this classifier was able to identify patients with F3/4 with an AUROC of 0.93.^108^ Shotgun sequencing of faecal metagenomes with molecular phenomics (hepatic transcriptome and plasma and urine metabolites) was conducted among a well‐characterized cohort of morbidly obese women. This study revealed molecular networks linking the gut microbiome and the host phenome to hepatic steatosis. Individuals with hepatic steatosis had low microbial gene richness and increased genetic potential for processing dietary lipids and endotoxin biosynthesis, hepatic inflammation, and dysregulation of aromatic and branched‐chain amino acid metabolism. These molecular phenomic signatures were predictive of hepatic steatosis (AUROC 0.87).^109^ Similar findings were noted in a twin‐family based study that used Magnetic Resonance Elastography (MRE) with proton density fat fraction (PDFF) to assess stage of hepatic fibrosis and grade of steatosis.^110^ Focusing on NASH cirrhosis based on MRE, a gut microbiome signature was identified among a cohort of 203 well‐characterized participants from a twin and family cohort. A panel of 30 features including 27 bacterial features was able to detect cirrhosis with an AUROC of 0.93.^111^ Taken together, these data suggest a role for the gut microbiome to help distinguish NAFL from NASH and to detect advanced fibrosis or cirrhosis in NASH. These results need to be further validated in larger, more diverse cohorts, however, before they can be applied in clinical practice.

## 6 SUMMARY

**FIGURE 2:**
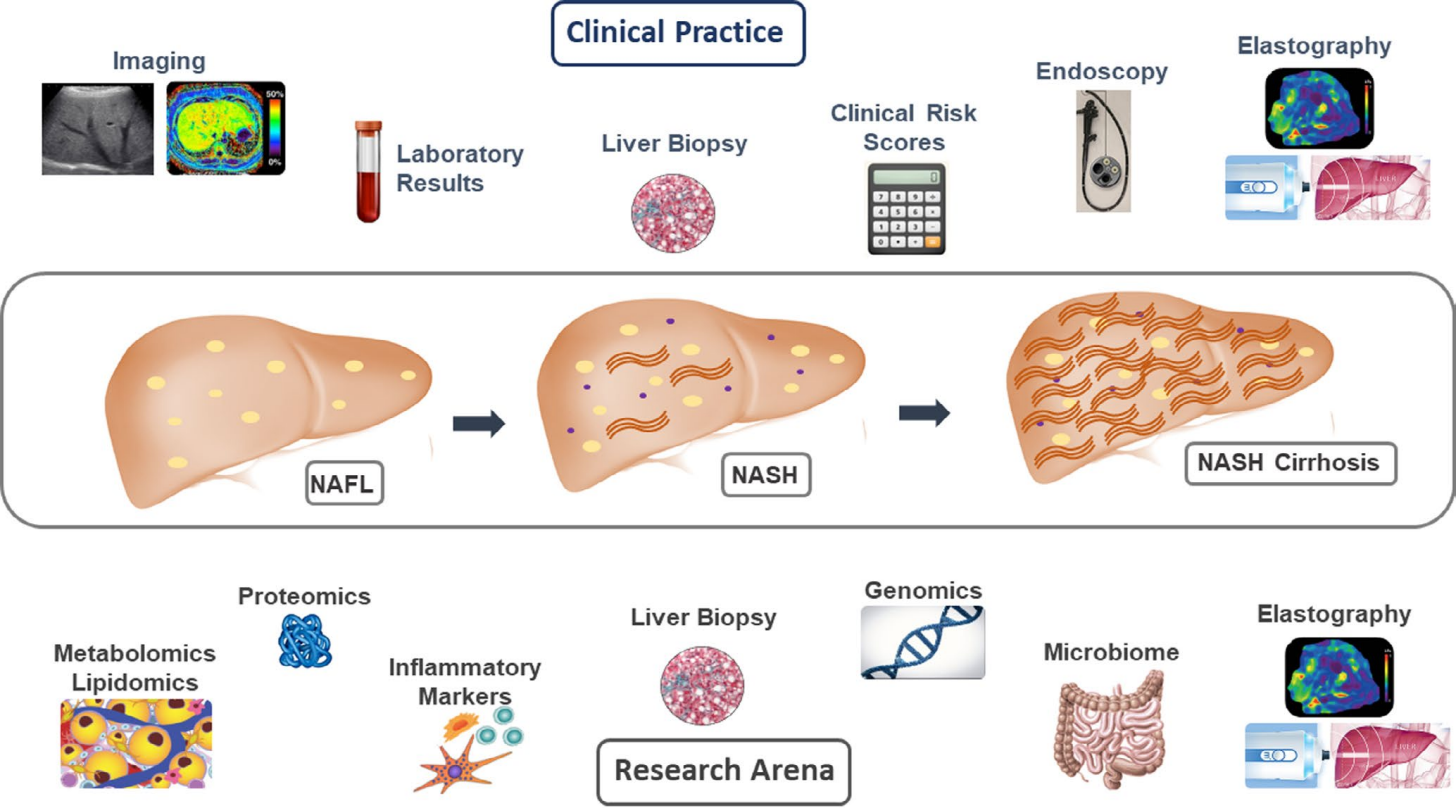
Approach to diagnosing and staging NASH: Clinical Practice compared to the research arena. Summary of categories of methods used for diagnosis and staging of NASH in clinical practice compared to those currently under investigation in the research arena

NAFLD is a significant global public health concern given its high prevalence and its associated morbidity and mortality. One of the central challenges to managing this burgeoning patient population is the difficulty in correctly differentiating individuals with NASH from the broader population of patients with NAFL. The other key barrier is identification of accurate, noninvasive methods to monitor response to treatment and disease progression. Presently, liver biopsy remains the gold standard method for diagnosis and staging of NASH. Histologic end‐points are also commonly used in the research arena for diagnosis and staging, including in NASH clinical trials. In clinical practice, liver biopsy is infrequently obtained however and providers rely on a combination of serum tests, imaging and endoscopic data for diagnosis and staging (Figure 2). Numerous diagnostic and interventional circulating biomarkers have been investigated to diagnose and stage NASH as outlined in this review. Several clinical decision aides using routinely available laboratory and clinical data have been validated to assess for risk of advanced fibrosis in NASH and can serve as useful initial risk stratification tools. The NFS and FIB‐4 provide high NPVs for likelihood of advanced fibrosis, but have limitations in terms of generalizability across age groups and categorization of 30% of individuals as having indeterminate scores. Serum biomarkers to assess necroinflammatory activity in NASH remain more challenging, though a number of combination panels have shown promising diagnostic accuracy. Emerging data suggest that incorporating novel approaches including genomics, proteomics and the gut microbiome may provide more individualized risk profiles that can better differentiate patients at higher risk of disease progression. Genomics data can potentially be used to assess risk for fibrosis progression and response to therapy and is likely to enter the clinical arena in the future.^76^, ^77^, ^78^, ^83^ Proteomics data have shown potential to differentiate NAFL from NASH, whereas lipidomics, metabolomics and the gut microbiome assessments have also been helpful in distinguishing stages of fibrosis in NASH.^65^, ^102^, ^103^, ^108^, ^109^ These ‘omics’ approaches require further validation in larger, more heterogeneous cohorts before they can be considered for use in clinical practice. Ongoing research suggests that combining circulating biomarkers with dynamic imaging modalities may yield better performance than using either modality alone. This combination approach likely represents a mechanism to improve our ability to noninvasively diagnose and monitor patients.

## CONFLICT OF INTEREST

The author has no conflicts of interest relevant to this manuscript.

## Data Availability

All data used in this review article are available upon request to the author or via open access journal data availability through cited article journal policies.
